# Metabolomic Changes in Biofilm of *Pseudomonas aeruginosa* Under Early-Stage Tobramycin Treatment Identified by 2D NMR-Based COLMARq

**DOI:** 10.1021/acs.jproteome.6c00323

**Published:** 2026-09-24

**Authors:** Munki Choo, Da-Wei Li, Kyungsuh Lee, Devin Sindeldecker, Lei Bruschweiler-Li, Rafael Brüschweiler

**Affiliations:** † Department of Chemistry and Biochemistry, 2647 The Ohio State University, Columbus, Ohio 43210, United States; ‡ Department of Microbial Infection and Immunity, The Ohio State University, Columbus, Ohio 43210, United States; § Department of Biological Chemistry and Pharmacology, The Ohio State University, Columbus, Ohio 43210, United States

**Keywords:** P. aeruginosa infection, Biofilm, Antibiotics, Metabolic adaptation, NMR-based metabolomics

## Abstract

Biofilms are estimated to account for a large proportion of persistent microbial infections and are associated with substantial mortality in hospitals. *Pseudomonas aeruginosa* biofilms exhibit higher resistance to antibiotics compared to their planktonic growth form, making them difficult to eradicate and contributing to the mounting global challenge posed by infectious diseases. To gain deeper insights into the metabolic response of biofilms to antibiotic pressure, we conducted a quantitative, untargeted metabolomics analysis using heteronuclear 2D NMR spectroscopy. Biofilms were grown in the presence of sub-inhibitory concentrations of tobramycin, and metabolic alterations were identified and quantified using the COLMARq analysis platform. Our analysis revealed significant metabolic shifts even at low doses of tobramycin with key metabolites such as UDP-glucose increasing and putrescine decreasing. Pathway enrichment analysis highlighted alterations in amino acid biosynthesis, energy metabolism, and stress response pathways. Notably, the accumulation of glutathione disulfide indicates enhanced oxidative stress, while the depletion of β-alanine, succinate, and GABA suggested metabolic shifts linked to biofilm adaptation. These findings provide new insights into the metabolic reprogramming of biofilms under antibiotic treatment and offer perspectives into biofilm behavior in the case of *Pseudomonas aeruginosa* when exposed to antibiotics from an early stage of infection.

## Graphical abstract

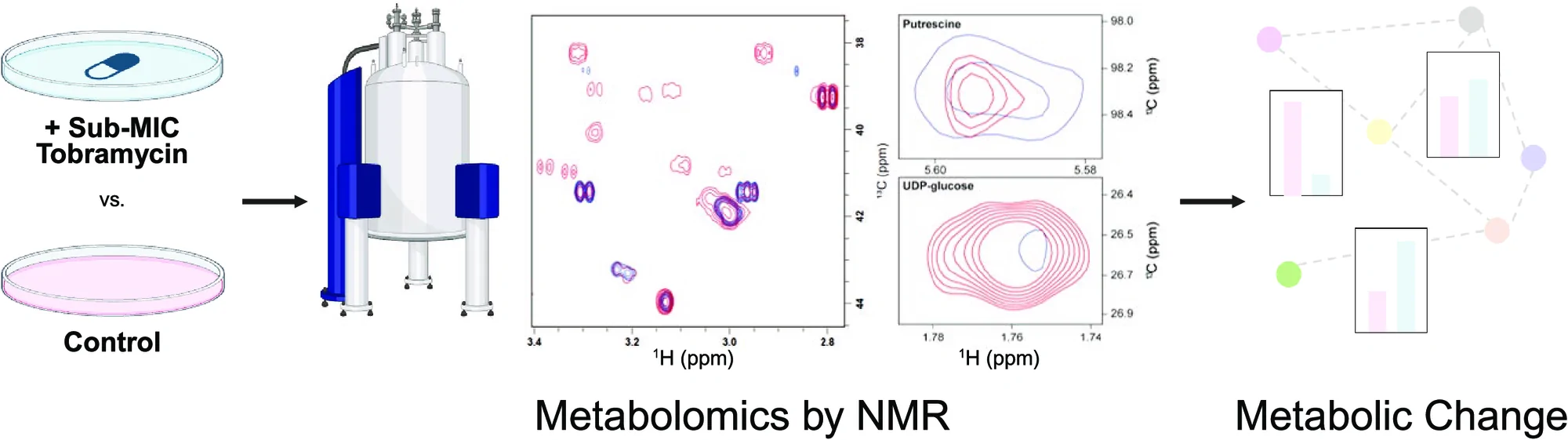

## 1 Introduction

Biofilms are highly structured bacterial communities embedded in an extracellular matrix, making associated infections difficult to eradicate.^1^ Compared to their planktonic counterpart, biofilms exhibit enhanced resistance to antibiotics, immune responses, and environmental stressors, contributing to chronic and recurrent infections.^2^ This is particularly concerning in implanted medical device-related infections,^3^ where biofilms protect bacteria from antibiotic penetration. Biofilms are not simply antibiotic-tolerant due to physical barriers; their metabolic flexibility plays a crucial role in their survival under antimicrobial stress.^4^ While genetic and proteomics studies have advanced our understanding of biofilm adaptation,^5^ metabolic changes in response to antibiotics remain less explored. Despite the routine use of antibiotics, biofilm-associated infections often persist and require surgical intervention and prolonged antimicrobial therapy. These clinical challenges underscore the need for a mechanistic understanding of biofilm responses to antibiotic exposure.

Among clinically relevant biofilm-forming pathogens, *Pseudomonas aeruginosa* is a major opportunistic pathogen responsible for chronic infections, particularly in immunocompromised individuals and patients with implanted medical devices.^6^ It is frequently associated with ventilator-associated pneumonia, cystic fibrosis-related lung infections, and catheter-related bloodstream infections. The intrinsic antibiotic resistance mechanisms and biofilm-forming ability of *P. aeruginosa* make its infections particularly difficult to treat, often necessitating combination antibiotic therapies and alternative treatment strategies.^7,8^ Biofilms persist despite antibiotic treatment due to their unique metabolic and physiological characteristics, yet the underlying mechanisms remain poorly understood.

*Pseudomonas aeruginosa* undergoes profound metabolic adaptations when transitioning from a planktonic to a biofilm lifestyle. At the cellular envelope, lipidome remodeling occurs; compared to planktonic cells, biofilm cells accumulate long-chain and cyclopropylated fatty acids while decreasing uneven-chain phospholipids, which stabilizes the lipid bilayer to withstand harsh microenvironments.^9^ Beyond structural changes, dynamic metabolic networks directly govern this lifestyle switch. Targeted molecular studies have highlighted the role of specific metabolic pathways in this transition; for instance, host-derived glycerol metabolism actively promotes biofilm formation by upregulating Pel exopolysaccharide production.^10^ Similarly, the biofilm-regulating second messenger cyclic dimeric guanosine monophosphate (c-di-GMP) is tightly linked to purine salvage, where specific environmental purines like adenosine and inosine can disrupt biofilm formation by decreasing c-di-GMP levels.^11^ While many studies have compared biofilms to their planktonic status, relatively few have examined metabolic differences between biofilms upon specific treatment. In particular, treatment-specific metabolic responses within established *P. aeruginosa* biofilms remain poorly characterized.

Among the antibiotics used to treat *P. aeruginosa* biofilms, tobramycin, a member of the aminoglycoside class, is widely utilized due to its strong bactericidal activity. It exerts its antimicrobial effect by binding to the bacterial 30S ribosomal subunit, leading to mistranslation and disruption of protein synthesis. Inhaled tobramycin formulations (e.g., TOBI®) are commonly used in the treatment of respiratory infections, including those in cystic fibrosis patients and individuals with chronic airway infections.^12^ Notably, sub-minimum inhibitory concentrations (sub-MICs) of antibiotics such as tobramycin do not always suppress biofilm formation and are frequently encountered in clinical settings.^13–15^ While aminoglycosides are highly effective against planktonic cells, their efficacy is significantly reduced in biofilms due to limited penetration, efflux pump activation, and altered metabolic states. Biofilms can undergo metabolic changes that help maintain their structural integrity and viability under antibiotic exposure, potentially contributing to increased tolerance.^16^ However, the specific metabolic pathways driving these changes remain unclear, highlighting the need for further metabolomic investigations.

Most biofilm metabolomic studies have been conducted using mass spectrometry (MS), a widely used technique known for its sensitivity and selectivity. While MS has contributed significantly to understanding biofilm metabolism,^17,18^ it has inherent limitations such as ion suppression and repeatability, adversely affecting quantification accuracy in complex biological samples.^19^ To complement these studies, we employed a nuclear magnetic resonance (NMR)-based untargeted metabolomics approach taking advantage of the highly quantitative and reproducible nature of NMR.^20^ Specifically, we used the COLMARq (Complex Mixture Analysis by NMR for quantification) web platform, enabling highly automated and fully quantitative analysis.^21^

Previous studies have extensively explored c-di-GMP, a second messenger that regulates the transition between planktonic and biofilm lifestyles in *P*. *aeruginosa*.^22^ High c-di-GMP levels promote biofilm formation, whereas low levels favor motility.^23^ Although its role in biofilm regulation is well-documented, biofilm formation involves multiple interconnected regulatory systems, including quorum sensing^24^ and matrix-associated pathways.^25^ Thus, its influence alone does not fully explain the metabolic shifts observed under antibiotic stress. Among metabolic factors linked to biofilms, polyamines such as putrescine contribute to stress responses, biofilm stability, and antibiotic resistance. Given the regulatory function of putrescine, investigating its role in the metabolic reprogramming of biofilms under antibiotic stress is crucial.

To address this, we applied NMR-based untargeted metabolomics to investigate metabolic changes associated with biofilm development under antibiotic stress from an early stage of infection. Our findings provide insight into the metabolic shifts that support biofilm survival under antibiotic stress, contributing to a better understanding of how metabolic interventions might assist biofilm persistence.

## 2 Materials and Methods

### Bacterial Strains, Growth Media, and Culturing Methods

*Pseudomonas aeruginosa* strain PAO1 ^26^ cultures were grown in lysogeny broth (LB) (Sigma-Aldrich), shaking at 220 rpm at 37 °C for 24 h to an optical density at 600 nm (OD_600_) of approximately 1.0. Cultures were diluted in LB to OD_600_ = 0.1, and 100 μL of the diluted suspension was plated on LB plates (28.4 cm^2^) containing 1.5% (w/v) agar. To examine the effects of tobramycin (Tokyo Chemical Industry), LB agar was supplemented with 0 (control), 0.5, 1, or 2 μg/mL of tobramycin prior to solidification. These concentrations were selected to be below the minimum inhibitory concentration (MIC) of tobramycin for PAO1, which is 4 μg/mL. Plates were incubated at 37 °C in 5% CO_2_ for 48 h, allowing biofilm formation in the presence of tobramycin.

### Metabolomics Sample Preparation

Biofilm cultures were harvested by scraping with a cell scraper (Fisher) and transferring the biomass into a microcentrifuge tube per sample and washed with chilled phosphate-buffered saline (PBS). Samples were immediately re-suspended in 600 μL cold 1:1 methanol (Fisher)/double distilled H_2_O (ddH_2_O) for quenching. A volume of 300 μL of 1.4 mm stainless-steel beads (SSB14B) was added, and cells were homogenized and lysed by a Bullet Blender (24 Gold BB24-AU by Next Advance) at a speed of 10 for 9 min at 4 °C.^27^ The sample was centrifuged at 14,000 × *g* for 20 min at 4 °C to remove beads and solid debris. The supernatant was transferred to a 15 mL conical tube, and 1:1:1 methanol/ddH_2_O/chloroform (Fisher) was added for a final volume of 7.44 mL.^28^ The sample was vortexed and centrifuged at 4,300 × *g* for 20 min at 4 °C for phase separation. After phase separation, the upper polar (methanol/water) phase was collected for NMR-based metabolomics and was carefully transferred into four microcentrifuge tubes per sample due to the limited tube capacity. The sample was centrifuged at 20,000 × *g* for 20 min at 4 °C to remove debris. The supernatant was transferred to a 15 mL conical tube, then dried with a vacuum concentrator (Thermo) and kept at −80 °C until further analysis. Before drying, 100 μL of each sample was saved for mass spectrometry (2.5% of total sample). For NMR measurements, the samples were re-suspended in 250 μL of NMR buffer (50 mM sodium phosphate buffer in D_2_O at pH 7.2) and centrifuged at 20,000 × *g* for 20 min at 4 °C for the removal of residual insoluble material/macromolecules (including proteins). The supernatant (210 μL) was carefully transferred to a new microcentrifuge tube, followed by the addition of 2.1 μL of 10 mM DSS (4,4-dimethyl-4-silapentane-1-sulfonic acid), used as an internal standard, to achieve a final concentration of 0.1 mM. The mixture was then transferred to a 3 mm NMR tube, capped with a polyethylene lid, and sealed with parafilm.

### NMR Experiments and Processing

NMR spectra were acquired at 298 K using a Bruker AVANCE III HD 850 MHz solution-state spectrometer equipped with a cryogenically cooled TCI probe. 1D ^1^H spectra (Bruker pulse program “noesygppr1d”) were obtained with 133,120 complex points during approximately 4 min. 2D ^1^H–^1^H TOCSY spectra were acquired (Bruker pulse program “dipsi2gpphzspr”) with 512 complex t_1_ and 8192 complex t_2_ points over a measurement time of about 4 h. The spectral widths along the indirect and direct dimensions were 12.0025 and 12 ppm, respectively, with 16 scans per t_1_ increment. 2D ^13^C–^1^H HSQC spectra (Bruker pulse program “hsqcetgpsisp2.2”) were collected with 1024 complex t_1_ and 4096 complex t_2_ points over 16 h. The indirect and direct dimensions’ spectral widths were 15.9816 and 160 ppm, respectively, with 32 scans per t_1_ increment. The 160 ppm ^13^C spectral width was selected to cover the main region of metabolite ^1^H–^13^C HSQC cross-peaks in the polar extracts analyzed in this study. The transmitter frequency offset values were 75 ppm in the ^13^C dimension and 4.705 ppm in the ^1^H dimension for experiments. NMR data were zero-filled twice in both dimensions, apodized using a cosine-squared window function, Fourier-transformed, and phase-corrected using the NMRPipe software.^29^

### NMR-Based Metabolomics Data Analysis

HSQC and TOCSY spectra were uploaded to the COLMARq web server for peak picking, peak alignment, metabolite identification, metabolite quantification via Voigt fitting, spectral normalization using a factor that is based on the average of the volume ratios with respect to a reference spectrum (±15% of all peaks right and left of the median), and univariate statistical analysis between cohorts.^21^ MetaboAnalyst was used to carry out multivariate statistical analyses.^30^ Metabolites were assigned to pathways using the KEGG PATHWAY database.^31^

### Statistical Analysis

All experiments were performed in at least three independent replicates. Two-tailed unpaired Student’s t-tests were performed to determine significant differences between groups. *p*-values below 0.05 were considered statistically significant. Error bars represent standard deviations. Pathway analysis was performed in MetaboAnalyst 6.0 using the KEGG pathway library for *Pseudomonas aeruginosa* PAO1. The Global Test was used for pathway-level p-values, and pathway impact was calculated using relative betweenness centrality. Pathways with Global Test p-values ≤ 0.05 were then ranked by pathway impact, and the top 10 pathways are reported.

### Bioinformatics Analysis

Gene Ontology (GO) enrichment analysis was carried out using DAVID bioinformatics resources^32,33^ and applied to the GSE65870 dataset. Biofilms that were not treated with tobramycin were chosen as the controls. Only the significantly different genes (*p*-value <0.05), as determined by Student’s t-test, were used for functional annotation. GO enrichment results were visualized using the Scientific and Research plot (SRplot) Web server.^34^

## 3 Results

### Untargeted Metabolomic Profiling and Multivariate Analysis of Biofilm Under Tobramycin Treatment

To investigate the global metabolic alterations induced by tobramycin treatment in *P*. *aeruginosa* biofilms, we conducted a quantitative, untargeted metabolomics analysis using 2D ^1^H–^13^C HSQC NMR spectroscopy, with metabolite identification and quantification performed via our public COLMARq web server.^21^ This untargeted approach allowed for the detection of a broad spectrum of metabolites without preselection based on prior knowledge, enabling an unbiased comparison of metabolic changes under different tobramycin concentrations. Representative 2D ^13^C–^1^H HSQC spectra of biofilms treated with different doses of tobramycin are shown in Figure 1A, revealing 883 distinct cross-peaks, indicative of the rich metabolic composition of these samples (expanded spectral regions are shown in Figures S1 and S2). The observed metabolic variations were further analyzed using unsupervised principal component analysis (PCA; Figure 1B). While PCA showed some overlap between the 1 μg/mL and 2 μg/mL groups, the control group (0 μg/mL) was clearly separated from all tobramycin-treated biofilms (0.5–2 μg/mL), indicating that even low-dose tobramycin induces systematic metabolic shifts in biofilms.

**Figure 1:**
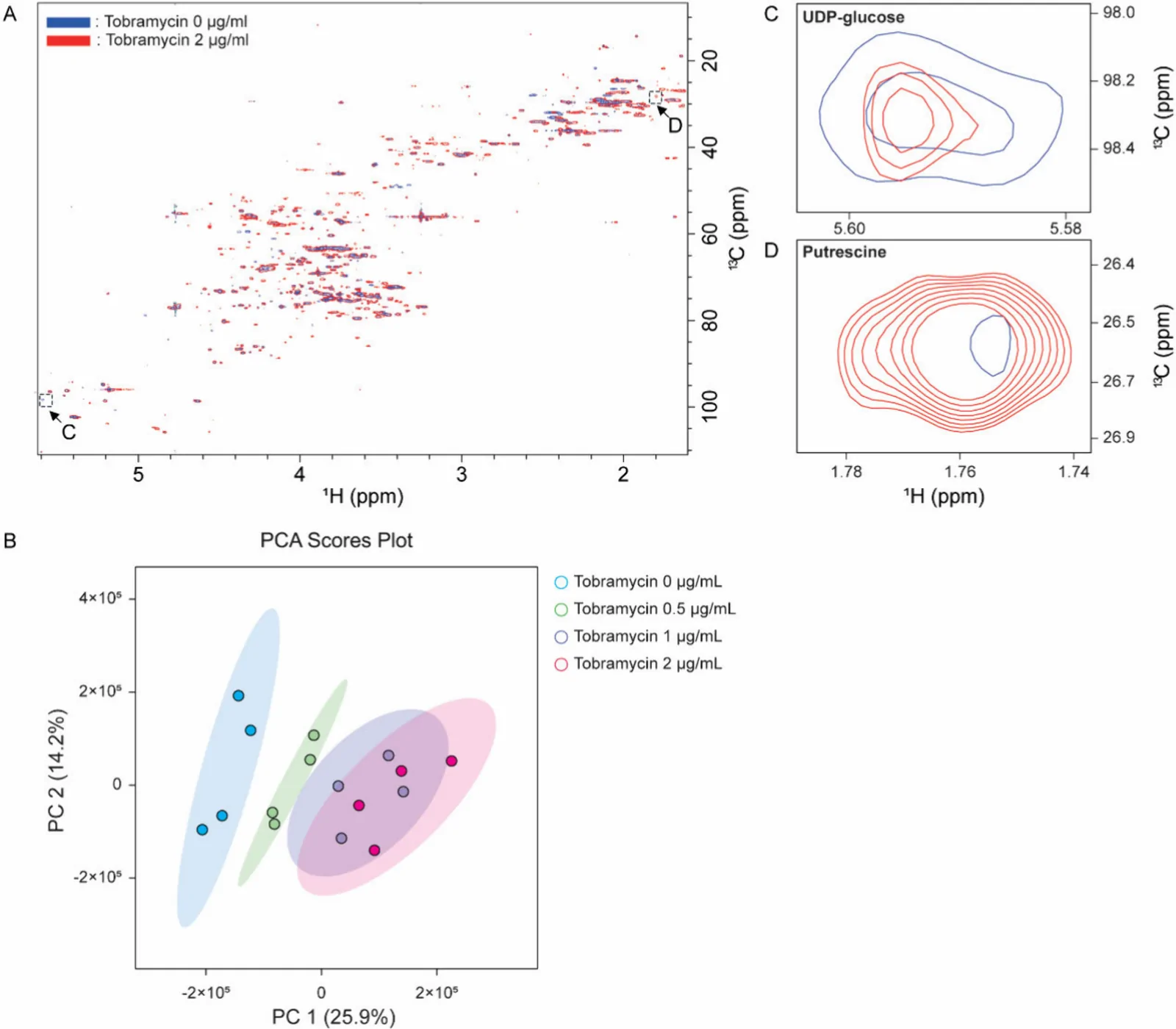
Representative NMR spectra and multivariate analysis of *Pseudomonas aeruginosa* biofilms under tobramycin treatment. (A) Overlay of representative 2D ^1^H–^13^C HSQC NMR spectra of biofilm extracts of *Pseudomonas aeruginosa* with 2 μg/mL tobramycin treatment (red) and without tobramycin treatment (blue) showing metabolite-specific changes. (B) Principal component analysis (PCA) score plot derived from NMR spectra (A) of biofilm with tobramycin. Ellipses indicate the 95% confidence interval for each group; colors denote tobramycin concentrations of 0 μg/mL (blue), 0.5 μg/mL (green), 1 μg/mL (purple), and 2 μg/mL (red). The percentages in the axis labels correspond to the percentages of variation covered by the corresponding principal components (PC). The dashed boxes in (A) correspond to UDP-glucose (C) and putrescine (D). The colors follow the same scheme as in panel (A).

### Metabolites Differing Significantly upon Tobramycin Treatment

Metabolomic analysis identified significant alterations in multiple metabolites upon tobramycin addition (Figure 2). Among these, uridine diphosphate glucose (UDP-glucose) exhibited a 5.76-fold increase, while putrescine was reduced to 3% of the control level (approximately 97% decrease; fold change = 0.03; *p* = 0.0051) (Figure 1C and 1D). Beyond these key metabolites, several other metabolic compounds exhibited smaller but statistically still significant changes, reflecting broader metabolic shifts. Notably, glutathione disulfide (GSSG) increased 3.94-fold, indicating an enhanced oxidative stress response. In contrast, metabolites involved in energy metabolism and amino acid biosynthesis, including β-alanine (0.26-fold), succinate (0.40-fold), gamma-aminobutyric acid (GABA) (0.46-fold), and aspartate (0.66-fold), were depleted.

**Figure 2:**
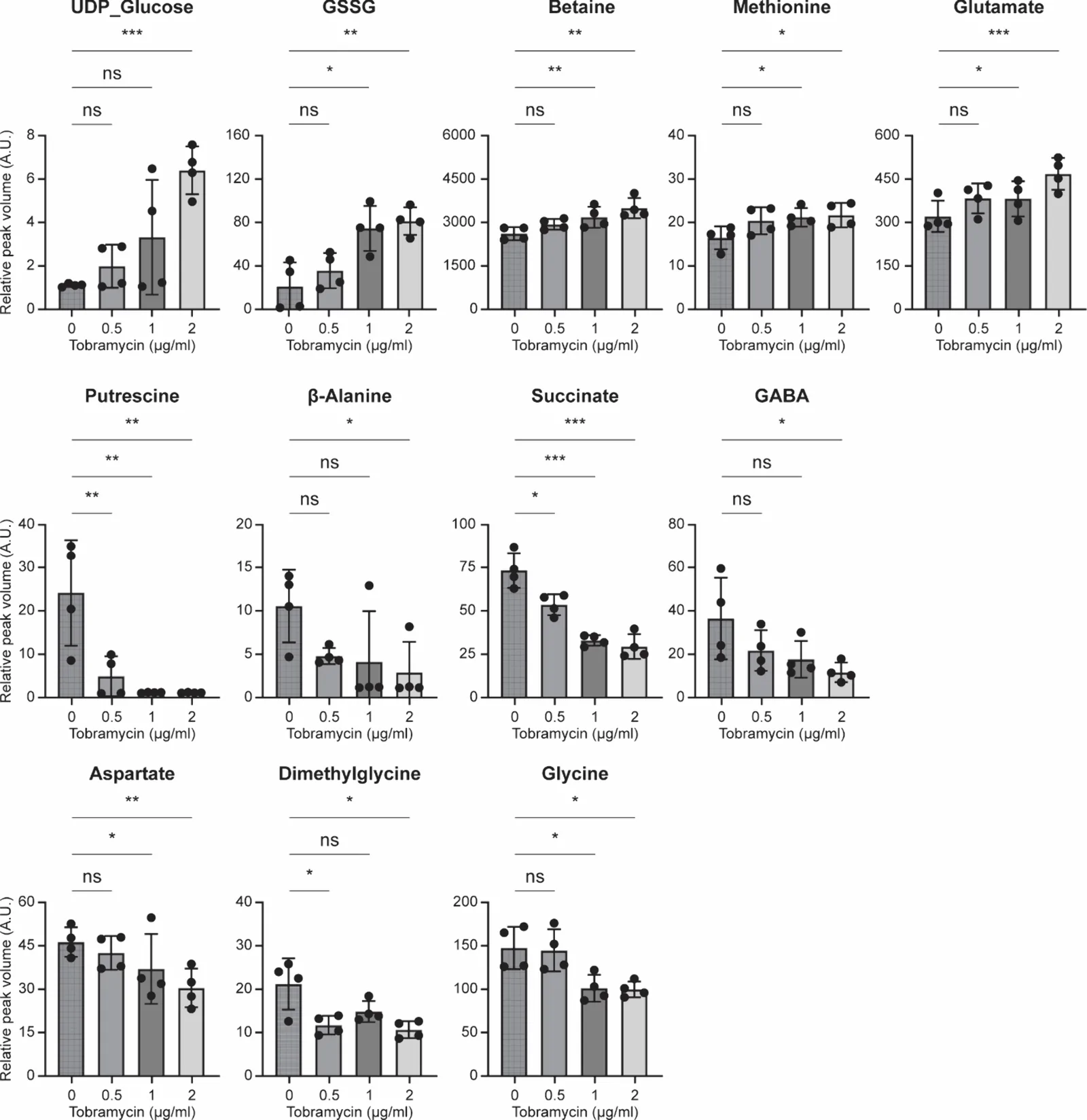
Levels of key metabolites in biofilm. The relative metabolite concentrations (*y*-axes) were determined by the normalized mean cross-peak volumes obtained from the 2D HSQC spectra in COLMARq (Figure 1A) (n = 4, each group). Data are presented as mean ± standard deviation (SD), with individual data points representing biological replicates. UDP-glucose was confirmed by spiking with an authentic standard (confidence level 1), whereas the other metabolites were assigned based on COLMAR reference matching supported by 2D NMR patterns (confidence level 2). The HSQC cross-peaks used for metabolite identification and quantification are shown in Supplemental Figure S1. Statistical comparisons between each tobramycin-treated group and the non-treated control (leftmost bar) were performed using unpaired t-tests. Significance is indicated as *p < 0.05, **p < 0.01, ***p < 0.001, or ns (not significant). Error bars represent standard deviations.

To further investigate these metabolic shifts, pathway enrichment analysis was conducted using MetaboAnalyst (Figure 3), revealing significant perturbations in multiple metabolic pathways. Using the KEGG pathway library for Pseudomonas aeruginosa PAO1, pathways with Global Test *p*-values ≤ 0.05 were selected and then ranked by pathway impact (relative-betweenness centrality); the top 10 pathways are reported in Figure 3 and Table 1. The top-ranked pathways based on pathway impact value included alanine, aspartate, and glutamate metabolism; glycine, serine, and threonine metabolism; β-alanine metabolism; and glutathione metabolism, among others. These pathways play essential roles in amino acid biosynthesis, energy production, redox balance, and stress response, respectively, all of which are crucial for biofilm survival under antibiotic pressure. Although polyamine biosynthesis was not directly enriched, the arginine and proline metabolism pathway (Figure 3, Table 1) may be linked to putrescine depletion since arginine serves as a precursor for putrescine synthesis. Key intermediates in this pathway, including l-proline, l-glutamate, putrescine, and GABA, were identified, suggesting metabolic alterations within this network. Additionally, the glutathione metabolism pathway suggests an enhanced oxidative stress response, further supported by the accumulation of glutathione disulfide (3.94-fold, Figure 2) under antibiotic pressure.

**Figure 3:**
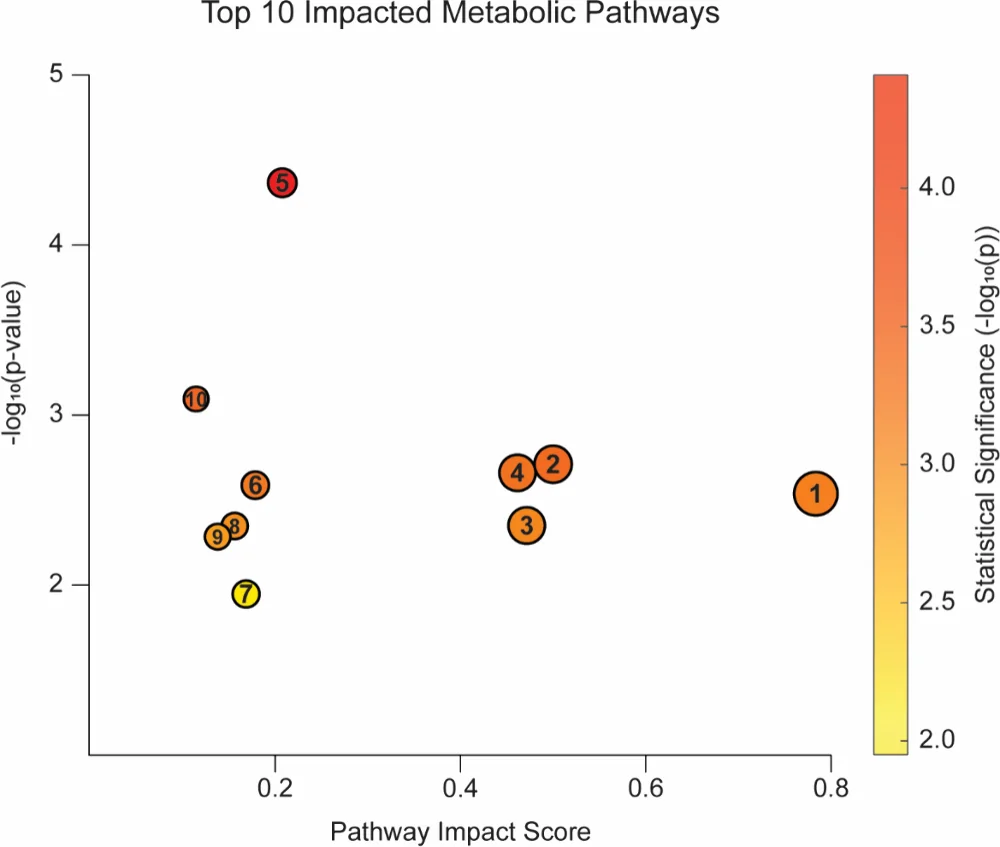
Pathway analysis of biofilms under tobramycin treatment with pathway impact scores and statistical significance. The circles with numbers belong to the following pathways: ① Alanine, aspartate and glutamate metabolism. ② Cyanoamino acid metabolism. ③ Glycine, serine and threonine metabolism. ④ Beta-alanine metabolism. ⑤ Glutathione metabolism. ⑥ Arginine and proline metabolism. ⑦ Methane metabolism. ⑧ Cysteine and methionine metabolism. ⑨ One carbon pool by folate. (Circled Number 10) Nicotinate and nicotinamide metabolism. The circle color is based on its *p*-value, and the radius is determined based on the pathway impact values (see Table 1). The color chart (varying from yellow to red) reflects different levels of significance in terms of its *p*-value.

**Table 1:** Pathway Analysis Results of *Pseudomonas aeruginosa* Biofilms under Tobramycin Treatment^a^

	Pathway Name	*p*-Value	Impact	Match Status	Matched Metabolites
1	alanine, aspartate and glutamate metabolism	2.90 × 10^–3^	0.78	6/22	aspartate, alanine, glutamate, 4-aminobutanoate, glutamine, succinate
2	cyanoamino acid metabolism	1.95 × 10^–3^	0.50	3/18	glycine, aspartate, serine
3	glycine, serine and threonine metabolism	4.46 × 10^–3^	0.47	6/37	glycine, aspartate, serine, threonine, glycine betaine, N,N-dimethylglycine
4	beta-alanine metabolism	1.29 × 10^–3^	0.46	3/15	beta-alanine, aspartate, 1,3-diaminopropane
5	glutathione metabolism	4.50 × 10^–5^	0.21	4/21	glutathione disulfide, glycine, glutamate, putrescine
6	arginine and proline metabolism	2.59 × 10^–3^	0.18	4/25	proline, glutamate, putrescine, 4-aminobutanoate
7	methane metabolism	1.13 × 10^–2^	0.17	2/26	glycine, serine
8	cysteine and methionine metabolism	4.49 × 10^–3^	0.15	3/45	methionine, aspartate, serine
9	one carbon pool by folate	1.95 × 10^–3^	0.14	3/13	glycine, serine, methionine, glycine betaine
10	nicotinate and nicotinamide metabolism	8.04 × 10^–4^	0.11	3/16	aspartate, succinate, nicotinate

a The table presents the top 10 impacted metabolic pathways, ranked based on their impact scores. The *p*-value represents the statistical significance of pathway enrichment, while the impact score is derived from pathway topology analysis, indicating the relative importance of the pathway based on matched metabolites. The match status column shows the number of detected metabolites compared to the total known metabolites in each pathway. The matched metabolites column lists the specific metabolites that were identified within each pathway. These pathway analysis results are visualized in Figure 3.

Building on these findings, transcriptomic analysis of biofilms under antibiotic stress (GSE65870) provided further insights into the molecular mechanisms driving these metabolic disruptions. Gene ontology enrichment analysis of differentially expressed genes (DEGs) identified biological processes closely linked to these pathway perturbations, including macromolecule metabolic processes, nucleic acid metabolism, and RNA metabolic processes. These findings suggest a transcriptionally regulated reprogramming of biofilm metabolism in response to tobramycin (Figure 4).

**Figure 4:**
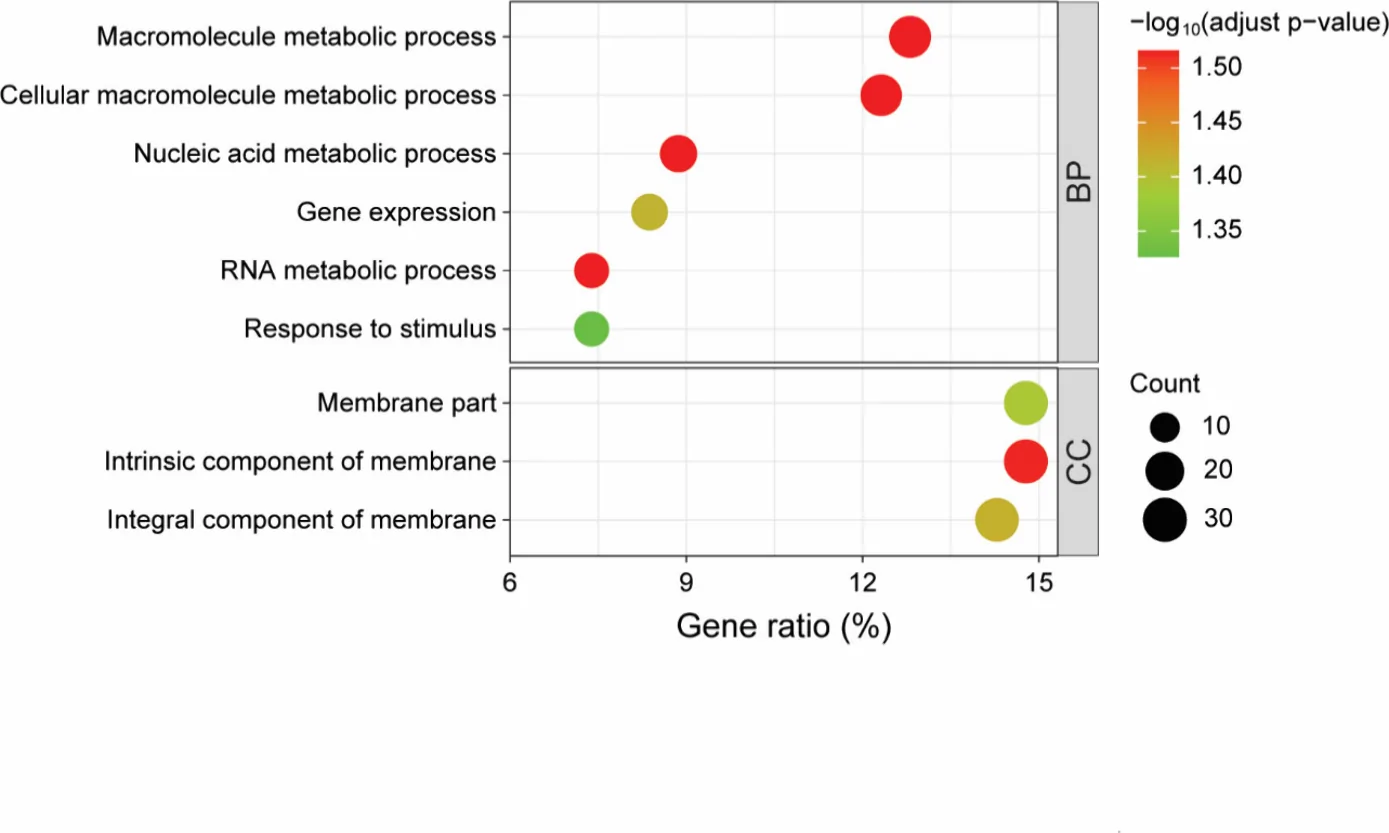
Gene Ontology (GO) analysis of genes affected by tobramycin in PAO1 biofilm. GO enrichment analysis was performed on differentially expressed genes (DEGs) with a cutoff of log_2_ fold change (FC) > 1.5 and adjusted *p*-value <0.05. The plot displays significantly enriched GO terms categorized under Biological Process (BP) and Cellular Component (CC). The *x*-axis represents the gene ratio (%) for each GO term, while the color scale denotes the −log_10_ (adjusted *p*-value), with red indicating more significant enrichment. The size of the circles corresponds to the number of genes associated with each term.

### Putrescine Supplementation Modulates Biofilm Metabolism and Enhances Glutamylation

Previous metabolomic analysis revealed a marked reduction in putrescine levels in biofilms treated with 2 μg/mL tobramycin (Figure 2, 97% decrease; fold change = 0.03). Given the known role of putrescine in polyamine metabolism, biofilm stability, and stress responses, we investigated whether putrescine supplementation could modulate biofilm metabolism and potentially counteract the metabolic shifts induced by tobramycin. To explore this, biofilms were supplemented with exogenous putrescine, and metabolic changes were analyzed. PCA revealed a clear separation between putrescine-supplemented and non-supplemented groups, indicating that putrescine supplementation led to distinct metabolic shifts in biofilms (Figure 5A). Putrescine supplementation led to significant metabolic changes, particularly in amino acid metabolism. Levels of glutamate and GABA increased (Figure 5B), suggesting a shift in glutamatergic metabolism. Additionally, increases were observed in metabolite signals associated with γ- glutamylation, a metabolic process linking putrescine metabolism with glutamate and GABA metabolism. Meanwhile, methionine, glycine, glycine betaine (N, N, N-trimethylglycine, commonly referred to as betaine), and dimethylglycine decreased. The depletion of methionine, glycine, and betaine suggests a potential impact on methylation and one-carbon metabolism, as these metabolites play key roles in methyl group donation and osmoprotection. While putrescine levels were restored, UDP-glucose levels declined, indicating potential alterations in extracellular polymeric substances (EPS) metabolism.

**Figure 5:**
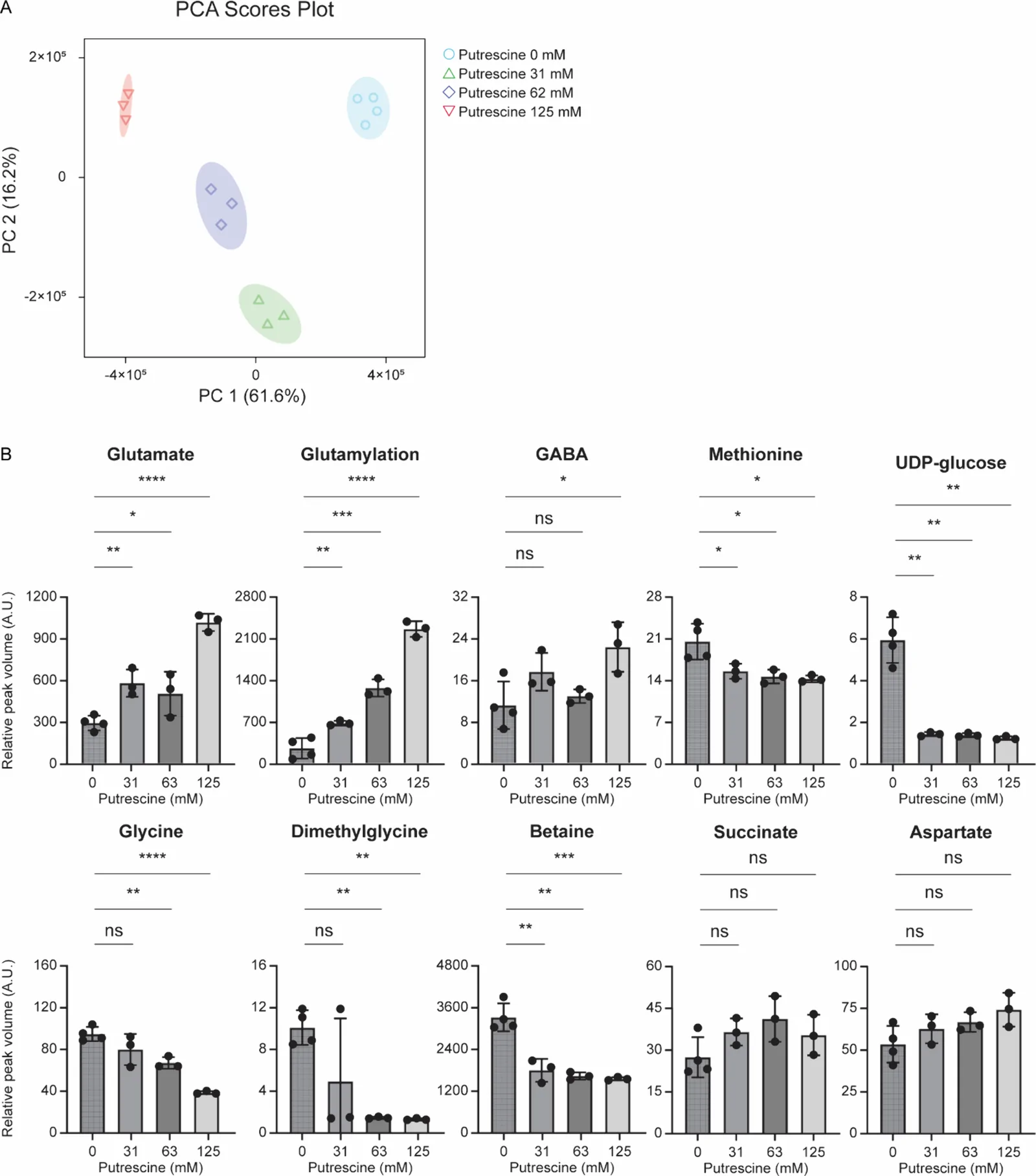
Metabolomic analysis of PAO1 biofilms supplemented with putrescine under tobramycin treatment. (A) Principal Component Analysis (PCA) score plot illustrates the metabolic variation among biofilms treated with different concentrations of putrescine (0, 31, 63, and 125 mM) with 2 μg/mL tobramycin present. Ellipses indicate the 95% confidence interval for each group; colors denote putrescine concentrations of 0 mM (blue), 31 mM (green), 62 mM (purple), and 125 mM (red). (B) Relative peak volumes of selected metabolites significantly affected by putrescine supplementation. Statistics are as in Figure 2. Glutamylated metabolites are described in the main text.

## 4 Discussion

### Novelty

Here, we characterized the impact of antibiotic exposure on specific metabolic pathways within *P. aeruginosa* biofilms and assessed how these metabolic changes contribute to biofilm adaptation. To this end, an NMR-based untargeted metabolomics approach was used to quantitatively analyze the metabolic changes associated with antibiotic administration in biofilms. Unlike post-treatment models, in our experiments, biofilms were allowed to develop from the onset in the presence of tobramycin. This experimental design differs from other protocols in which antibiotics are applied to preformed biofilms and instead allows us to observe metabolic characteristics that emerge during biofilm development under sub-inhibitory antibiotic exposure. Therefore, the metabolic changes observed in this study do not represent classical responses of adaptation to antibiotic stress but rather reflect the developmental trajectory of biofilms forming in an antibiotic environment. Most existing studies have characterized biofilms by comparing biofilm and planktonic states. These studies have focused on identifying physiological differences between the two states and have been used to understand how the transition from planktonic to biofilm state responds to environmental changes.^35^ In addition, biofilms have been characterized primarily at the gene expression and protein level, but there is a lack of studies that directly address metabolic regulation.^36^ Therefore, this study is notable in that it focuses on how biofilms undergo metabolic changes in an antibiotic environment, i.e., rather than simply analyzing structural changes or growth differences, to address a key question about metabolic regulation in the response of biofilms to antibiotic stress.

## Explanation of Changes in Metabolites

The metabolic changes in *P. aeruginosa* biofilms upon tobramycin treatment suggest that biofilms undergo metabolic remodeling in response to the antibiotic environment through metabolic regulation, well beyond a mere physical barrier. The observed metabolic changes are associated with EPS synthesis, energy metabolism, polyamine metabolism, and oxidative stress regulation, providing a more specific understanding of factors that may help biofilms survive in an antibiotic-stressed environment.

One of the most prominent changes was an increase in UDP-glucose. UDP-glucose is a major precursor for EPS biosynthesis,^37^ and biofilms are known to increase EPS production under antibiotic exposure. EPS are central components of the biofilm matrix and are composed of biopolymers such as polysaccharides, proteins, and nucleic acids; EPS can constitute up to 90% of the total organic matter per unit biofilm volume. Importantly, EPS have multiple protective functions in pathogenic biofilms, including restricting the penetration of antibiotics and other molecules.^38^ These findings suggest a reorientation of carbon metabolism toward EPS associated pathways, closely linked to alterations in the tricarboxylic acid (TCA) cycle, a central component of carbon metabolism.

The main metabolites involved in the TCA cycle, succinate and aspartate, showed a decrease. The decrease in succinate may reflect modulation of the TCA cycle under antibiotic stress. Aspartate acts as a key metabolite in several amino acid and nitrogen metabolism pathways^39,40^ and was also found to decrease. Pathway enrichment analysis^41^ showed that the alanine, aspartate, and glutamate metabolism pathways were the most affected (Rank 1, Figure 3). However, it is unclear if these metabolic changes are simply due to the regulation of enzyme activity or if they are also regulated at the gene expression level. To further analyze this, we performed a Gene Ontology analysis and found that the term “macromolecule metabolic process,” which encompasses the biosynthesis and modification of proteins, nucleic acids, and lipids, was significantly altered (Figure 4). Notably, several more specific subcategories such as “nucleic acid metabolic process”, “RNA metabolic process”, and “gene expression” were also enriched (Figure 4), suggesting that the observed metabolic changes may reflect broader transcriptional regulation.

These changes also involve polyamine metabolism. Indeed, a decrease in putrescine was seen under antibiotic stress. In the metabolic pathways associated with putrescine, the concentration of glutamate tended to increase, while GABA synthesized from glutamate tended to decrease. These findings indicate that glutamate may have been linked to other metabolic pathways (e.g., oxidative stress response) rather than GABA synthesis. Interestingly, glutamate acts as a precursor to glutathione, which is closely related to the regulation of oxidative stress.^42^ In fact, antibiotics are known to induce oxidative stress within cells,^43–46^ and defense mechanisms against oxidative stress play an important role in bacterial survival and stress response. To overcome this oxidative stress, biofilms possess redox systems, the main one being reduced glutathione (GSH) and oxidized glutathione (glutathione disulfide, GSSG). In this study, an increase in GSSG was identified, which is consistent with enhanced oxidative stress-related processes under antibiotic exposure. Pathway enrichment analysis showed that the glutathione metabolism pathway was altered (Pathway 5, Figure 3). These findings support the role of oxidative stress regulation as an integral part of biofilm response to antibiotic stress.

As a result, biofilms appear to adjust their survival responses in the antibiotic environment through complex metabolic regulatory mechanisms that include regulation of energy metabolism, regulation of putrescine metabolism, and oxidative stress response, in addition to physical defense mechanisms. This metabolic regulation was further clarified by pathway enrichment analysis.

## Putrescine Supplementation

Putrescine is a polyamine that plays an important role in the regulation of growth and metabolism, maintaining cell stability, and intracellular pH homeostasis, among other functions.^47,48^ In addition, previous studies have shown that putrescine can promote biofilm formation and increase intracellular c-di-GMP levels in *P. aeruginosa* under non-antibiotic conditions.^49^ However, polyamines like putrescine can also increase membrane permeability, thereby enhancing the bacterium’s susceptibility to antibiotics.^50^ Consistent with this vulnerability, in the present study, putrescine concentrations decreased upon antibiotic treatment. It can be inferred that putrescine may function as a metabolically active intermediate under antibiotic stress, potentially associated with broader metabolic adjustments. Therefore, to assess whether the decrease in putrescine reflects regulated metabolic changes or simply a general stress response, we analyzed the metabolomic changes in a separate experiment (Figure 5).

After putrescine supplementation, an increase in glutamate and GABA concentrations was observed, with a concomitant increase in glutamylation-related metabolites. This indicates a metabolic connection between polyamine and glutamate pathways. A study reported on *Escherichia coli* confirmed that putrescine can be converted to GABA via γ-glutamylation, highlighting this process as a potential link between polyamine and glutamate metabolism and its integration into the TCA cycle.^51^ The observed increase in glutamylation-related metabolites suggests that GABA-associated metabolic processes are present in *P. aeruginosa*. Beyond serving as a metabolic intermediate, GABA holds a profound physiological role in P. aeruginosa biology and biofilm regulation. Specifically, GABA has been identified as a specific virulence regulator in P. aeruginosa, where its presence directly influences biofilm adhesion properties and triggers the overexpression of oxygen-scavenging proteins to adapt to restricted microenvironments.^52^ Under severe environmental pressures, such as antibiotic exposure, bacteria actively reprogram these intricate stress-response networks to prioritize immediate survival and antibiotic tolerance.^53^

This suggests that biofilms adjust putrescine levels while coordinating the metabolic balance with the glutamate and TCA cycles. This extends the link between polyamine metabolism and glutamate metabolism reported in previous studies and suggests that polyamine-associated pathways in biofilms may be linked to broader metabolic coordination beyond structural roles. Notably, succinate and aspartate levels, which were reduced by tobramycin treatment, both increased after putrescine supplementation; however, these changes were not statistically significant. This suggests that putrescine may contribute to the partial recovery or stabilization of the TCA cycle and amino acid metabolism through glutamate-related pathways.

Under tobramycin treatment, we observed significant changes in both UDP-glucose and putrescine. Rather than reflecting a direct metabolic link, these changes highlight a co-occurring metabolic remodeling driven by antibiotic stress. Specifically, UDP-glucose, a primary precursor for exopolysaccharide biosynthesis, exhibited a 5.76-fold increase. Since exopolysaccharide forms the protective physical barrier of the biofilm matrix, this accumulation may suggest that the cells actively reroute carbon flux to reinforce their structural defense against tobramycin. Concurrently, putrescine, a polyamine intricately linked to cellular stress responses, was severely depleted. Interestingly, when exogenous putrescine was supplied, UDP-glucose levels exhibited a decreasing trend. These observations are consistent with a coordinated response to early antibiotic stress, involving increased EPS-precursor synthesis and changes in polyamine metabolism. The addition of exogenous putrescine may alleviate the overall stress state of the biofilm, thereby reducing the compensatory need to EPS precursors like UDP-glucose.

### Limitations

As demonstrated in this study, the use of PAO1 on agar is well-suited for quantitative biofilm metabolomics studies. For more clinically oriented research, these studies can be extended, for example, to cystic fibrosis sputum or polymicrobial biofilms. Although many of the observed metabolic changes are statistically significant (p < 0.05), their correlative nature does not allow unambiguous establishment of underlying causalities. In order to further validate the functional role of metabolic changes observed here, future research should focus on the following areas: First, the possibility of modulating biofilm resistance through modulation of polyamine metabolism should be examined. Although this study focused on metabolism, further gene expression analysis and proteomics studies are needed to determine if and how these changes are linked to specific genes and enzymes. Second, comparative studies are needed to determine if the changes in putrescine metabolism are specific to tobramycin or if similar patterns are seen with other classes of antibiotics, such as β-lactam or quinolone. By analyzing changes in the biofilm metabolome under different antibiotic treatment conditions, the role of putrescine metabolism in biofilm-associated antibiotic responses can be further refined.

## Conclusion

In conclusion, metabolic changes in *P. aeruginosa* biofilms upon early tobramycin treatment were analyzed using a fully quantitative, untargeted 2D NMR-based metabolomics approach based on COLMARq, and significant changes in several metabolic pathways were identified. This highlights the involvement of metabolic changes in biofilm-associated antibiotic resistance and suggests that putrescine and its metabolic regulation represent viable targets for further investigation. Future studies may further elucidate the relationship between putrescine metabolism and biofilm resistance, which could lead to the exploration of novel anti-biofilm strategies.

## Supplementary Material



## Data Availability

The raw NMR data and associated metadata used in this study have been deposited in the Zenodo repository under the DOI: 10.5281/zenodo.19755447.

## Notes

Figure S1 - Representative NMR spectra of biofilm extracts with tobramycin treatment; Figure S2 - Representative NMR spectra of biofilm extracts without tobramycin treatment (PDF)

## ⊗

Department of Paramedicine, Kyungil University, Gyeongsan, 38428, Republic of Korea
