# Supplementary material for: Metabolomic Changes in Biofilm of Pseudomonas aeruginosa Under Early-Stage Tobramycin Treatment Identified by 2D NMR-Based COLMARq
Source: J Proteome Res. 2026 Sep 24;25(11):5873–83. doi: 10.1021/acs.jproteome.6c00323 (PMC13750080; doi:10.1021/acs.jproteome.6c00323)
Supplement: Supplementary file 1 [file pr6c00323_si_001.pdf]

SUPPORTING INFORMATION

Metabolomic Changes in Biofilm of Pseudomonas aeruginosa Under Early-Stage Tobramycin Treatment Identified by 2D NMR-based COLMARq

Munki Choo[1, a*], Da-Wei Li[1], Kyungsuh Lee [1], Devin Sindeldecker[2], Lei Bruschweiler-Li[1], and Rafael Brüschweiler[1, 3*]

[1]Department of Chemistry and Biochemistry, The Ohio State University, Columbus, Ohio 43210, U.S.A.

[2]Department of Microbial Infection and Immunity, The Ohio State University, Columbus, Ohio 43210, U.S.A.

[3]Department of Biological Chemistry and Pharmacology, The Ohio State University, Columbus, Ohio 43210, U.S.A.

[a]Current Address: Department of Paramedicine, Kyungil University, Gyeongsan, 38428, Republic of Korea

* To whom correspondence should be addressed:

Dr. Munki Choo E-mail: mkchoo.lab@gmail.com

Dr. Rafael Brüschweiler E-Mail: bruschweiler.1@osu.edu

Content:

- **Figure S1:**
  Representative NMR spectra of biofilm extracts with tobramycin treatment (2 μg/mL of tobramycin)
- **Figure S2:**
  Representative NMR spectra of biofilm extracts without tobramycin treatment (0 μg/mL tobramycin)

Figure S1. Representative NMR spectra of biofilm extracts with tobramycin treatment (Tobramycin 2 µg/mL)

(A) Overview region of a representative 2D ^{1}H–^{13}C HSQC spectrum (^{1}H, 0–10 ppm; ^{13}C, 0–155 ppm) of a tobramycin-treated sample. The two black boxes in (A) indicate the regions that are shown as expansions in (B) and (C). (B) Expansion of the aliphatic region (^{1}H, 1.0–3.1 ppm; ^{13}C, 14–43 ppm). (C) Expansion of the $C_{\alpha}H$ and carbohydrate region (^{1}H, 2.6–6.0 ppm; ^{13}C, 41–107 ppm). Labeled cross-peaks indicate assigned metabolites.

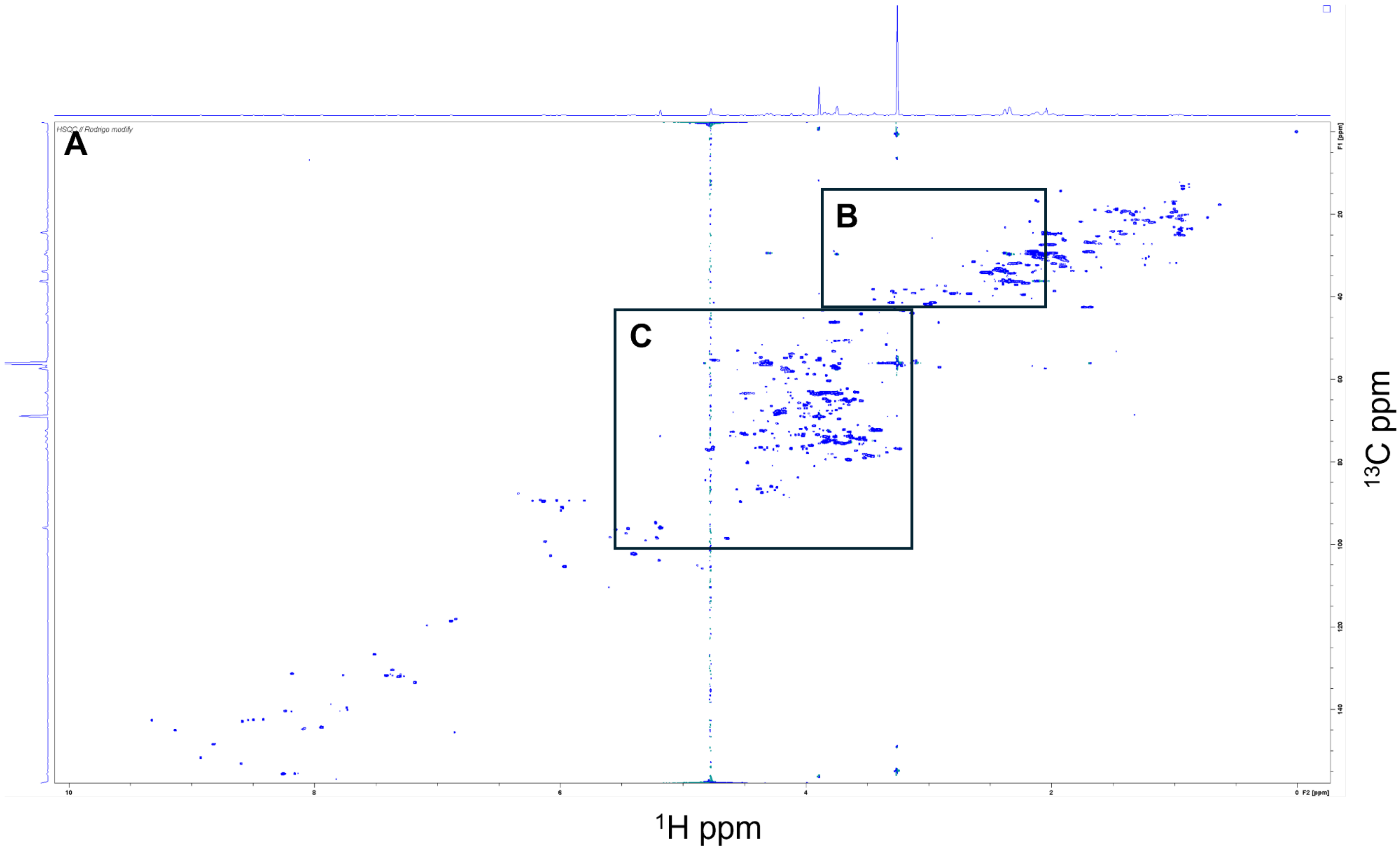
A
B
C
^{13}C ppm
^{1}H ppm

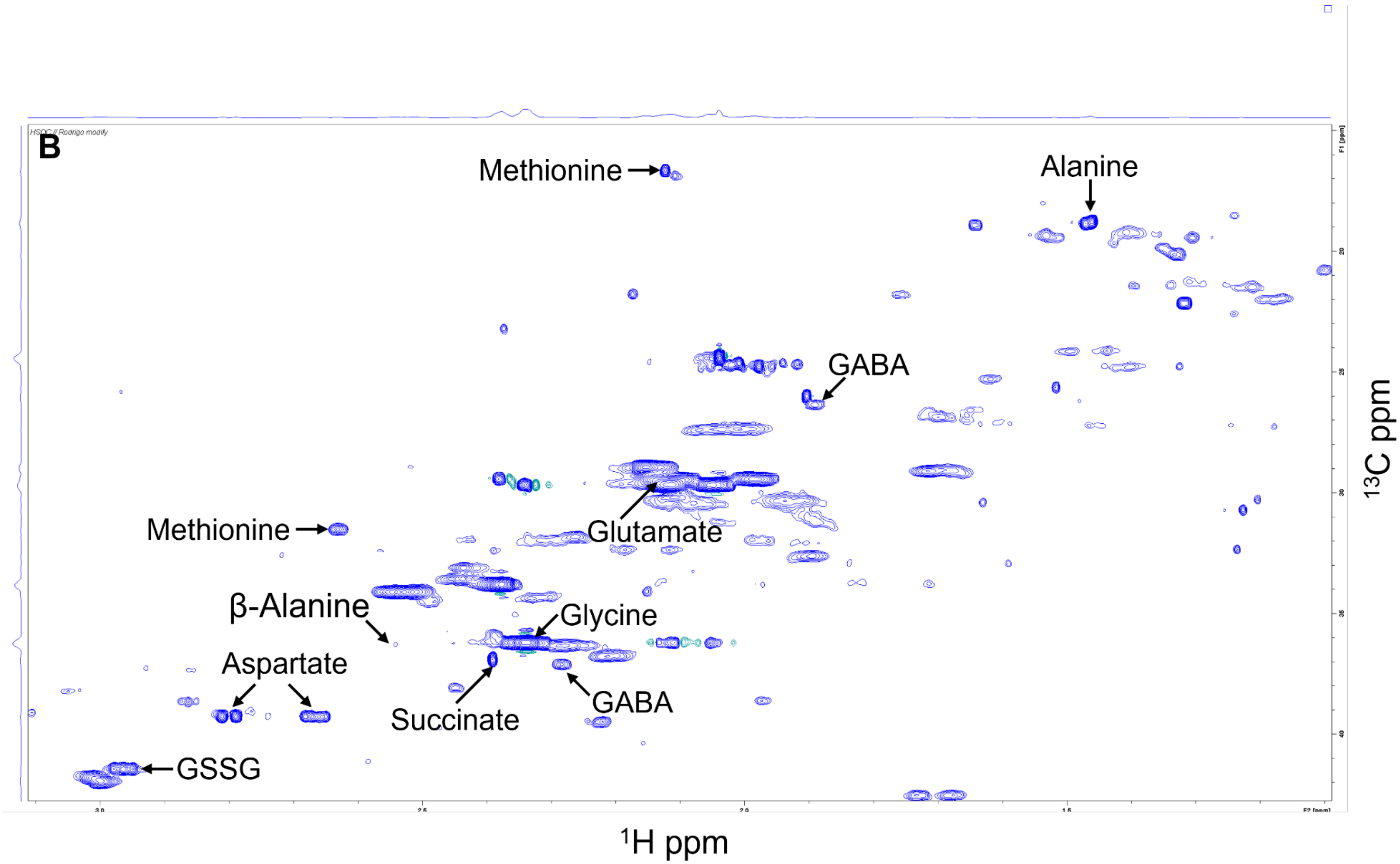

B
Methionine
Alanine
GABA
Glutamate
Methionine
β-Alanine
Glycine
Aspartate
Succinate
GABA
GSSG
^{13}C ppm
^{1}H ppm

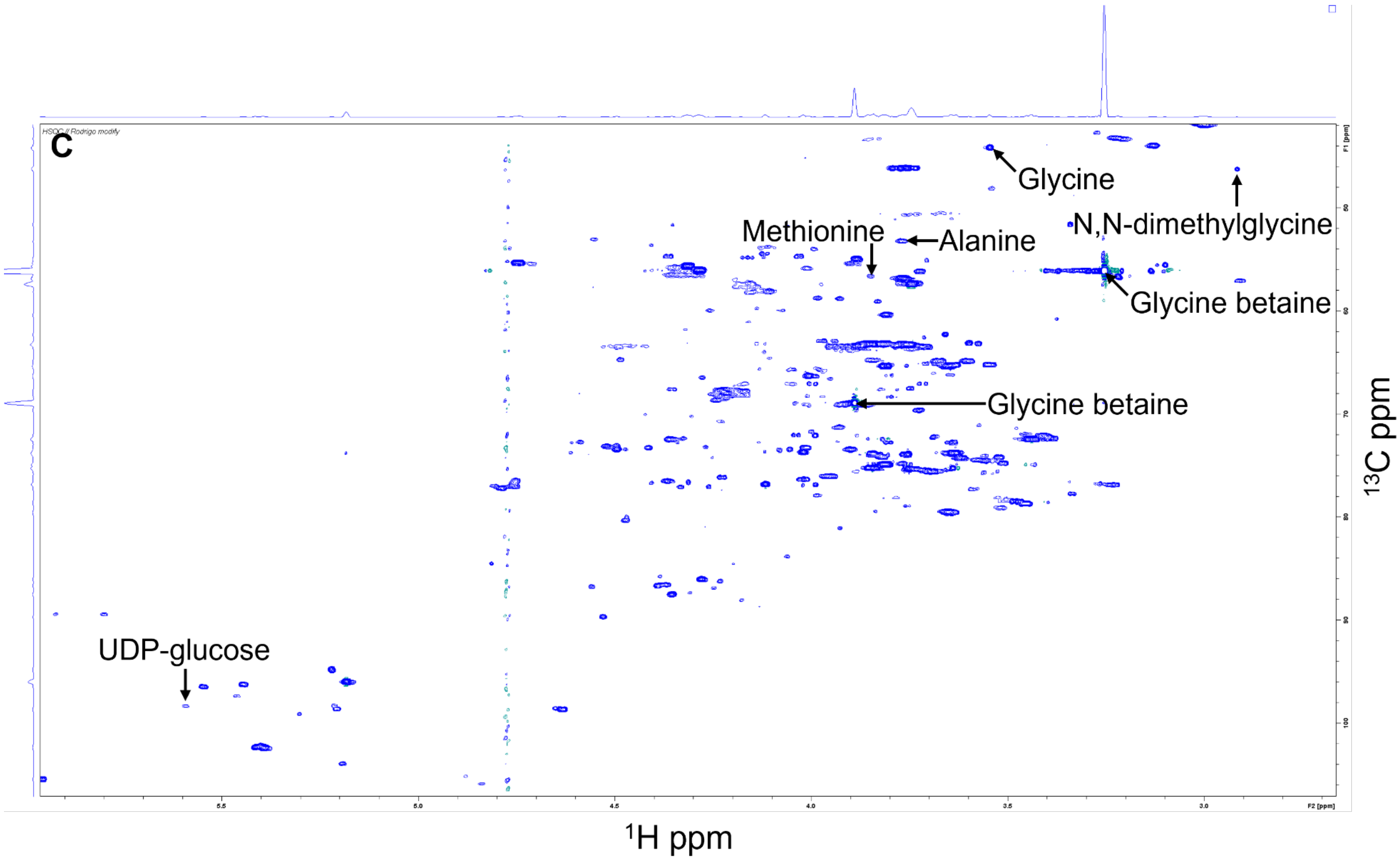
C
HSQC // Rodrigo modify
Glycine
N,N-dimethylglycine
Methionine
Alanine
Glycine betaine
Glycine betaine
UDP-glucose
5.5
5.0
4.5
4.0
3.5
3.0
F2 [ppm]
^{1}H ppm
^{13}C ppm

Figure S2. Representative NMR spectra of biofilm extracts without tobramycin treatment (Tobramycin 0 μg/mL)

(A) Overview region of a representative 2D ^{1}H–^{13}C HSQC spectrum (^{1}H, 0–10 ppm; ^{13}C, 0–155 ppm) of a sample in the absence of tobramycin. The two black boxes in (A) indicate the regions that are shown as expansions in (B) and (C). (B) Expansion of the aliphatic region (^{1}H, 1.0–3.1 ppm; ^{13}C, 14–43 ppm). (C) Expansion of the C$_\alpha$H and carbohydrate region (^{1}H, 2.6–6.0 ppm; ^{13}C, 41–107 ppm). Labeled cross-peaks indicate assigned metabolites.

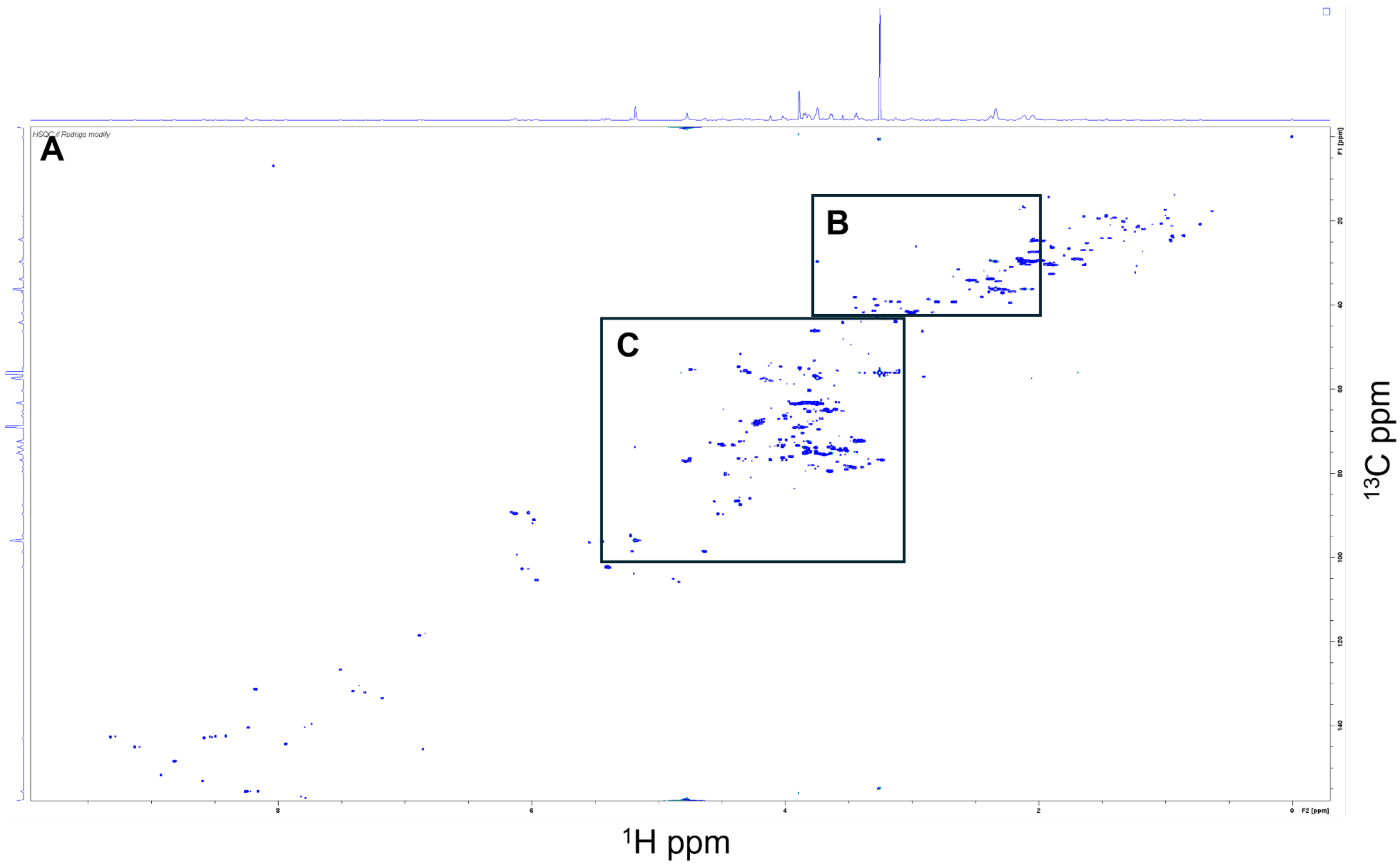

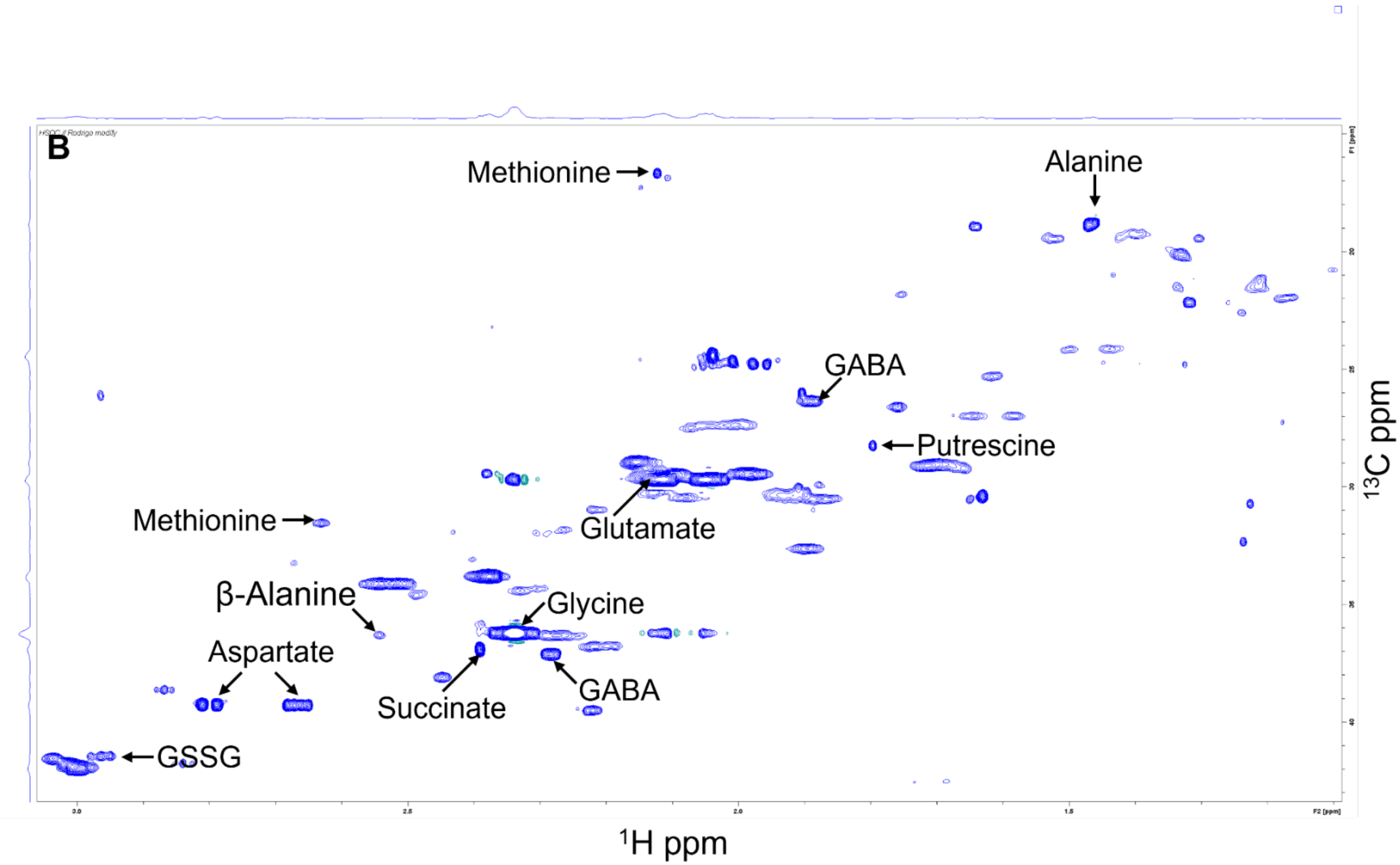
B
Methionine
Alanine
GABA
Putrescine
Methionine
Glutamate
β-Alanine
Glycine
Aspartate
Succinate
GABA
GSSG
¹H ppm
¹³C ppm

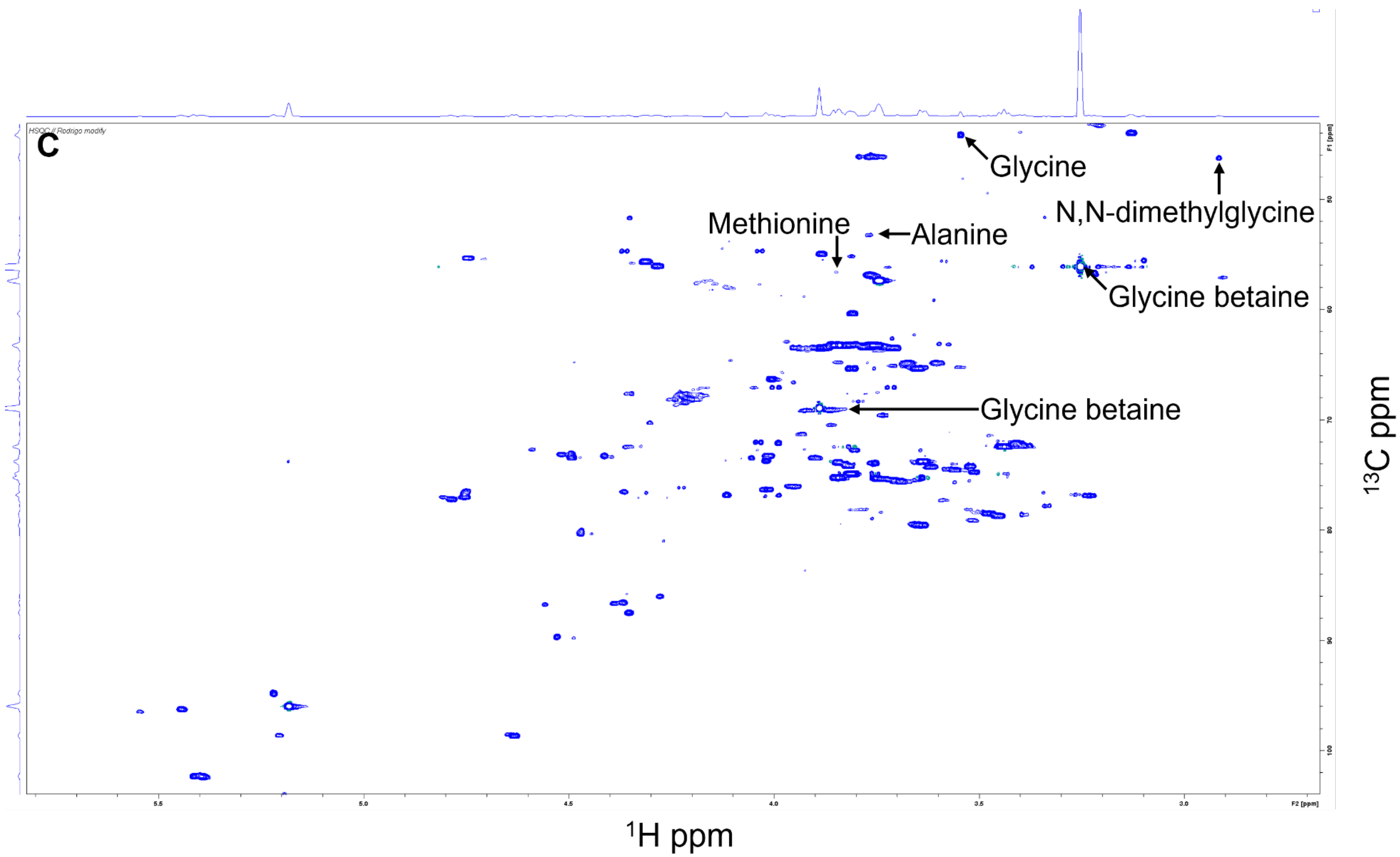
C
HSQC // Rodrigo modify
Glycine
N,N-dimethylglycine
Methionine
Alanine
Glycine betaine
Glycine betaine
^{13}C ppm
^{1}H ppm
